# Antibiotics in septoplasty: is it necessary?

**DOI:** 10.1016/S1808-8694(15)31241-6

**Published:** 2015-10-20

**Authors:** Marcello Caniello, Gustavo Haruo Passerotti, Elder Yoshimitsu Goto, Richard Louis Voegels, Ossamu Butugan

**Affiliations:** Physician; PhD. Fellowship in Endonasal Endoscopic Surgery – Otorhinolaryngological Department – Clinical Hospital - Medical School - USP; Intern Physician, Department of Otorhinolaryngology – Clinical Hospital – Medical School – USP; Physician; Ph.D. in Otorhinolaryngology, Medical School – USP; Physician, Associate Professor of Otorhinolaryngology, Medical School – USP; Physician, Associate Professor of Otorhinolaryngology, Medical School – USP

**Keywords:** septoplasty, antibiotics, infection, complications

## Abstract

The use of antibiotics is a common practice among otorhinolaryngogists for surgical procedures. The majority of the American Rhinology Society members uses postoperative antibiotics routinely in septoplasties, which is considered unnecessary by many authors.

**Aim:**

To study the real necessity of the antibiotic usage in septoplasties, as well as the main post-operative complications described in the literature.

**Study design:**

clinical prospective with transversal cohort.

**Material and Method:**

We studied prospectively 35 patients who were undergone to septoplasty with or without turbinectomy, in the Clinical Hospital of the University of São Paulo. The patients were splited in three groups: Group A: without antibiotics; Group B: antibiotics (cefazolin) only during the anesthesical induction; Group C: antibiotics both in the anesthesical induction and post-operatively for seven days. A questionnaire was applied in the immediate post-operatory, in the 7th post-operative day and in the 30th post-operative day asking for bleeding, fever, pain, nauseas, vomits and followed by physical and endoscopic evaluation looking for hematoma, septal abscess and purulent secretion which as quantified.

**Result:**

We do not observed significative difference among the groups concerning to pain, fever, nauseas, vomits, bleeding and purulent secretion. None of the patients had hematoma or septal abscess. The groups also do not differ in respect to the quantity of purulent secretion.

**Conclusion:**

The nasal surgeries are clean contamined and do not need antibioticprophilaxy because of the low infection risk.

## INTRODUCTION

The use of antibiotics in otorhinolaryngological surgeries is a common practice among most ENT doctors, however there are few studies proving the efficacy and need for this practice, which is considered as unnecessary by some authors3., 4., 5., 6., 7..

A survey carried out among the Members of the US Rhinology Society showed that 66% out of 448 doctors that answered the questionnaire used antibiotics as a routine practice in postoperative period of septoplasties1.

The surgical procedures of airways/digestive tract are potentially considered as contaminated^8^ and may be associated with postoperative infectious complications. Severe complications are described after septoplasties such as toxic shock syndrome (TSS), endocarditis, osteomyelitis, meningitis and cavernous sinus thrombosis (CST), the latter are fortunately extremely rare, though^3^^,^^9^.

Makitie et al.^3^ reported postoperative infection in 12% of 100 patients that have undergone septoplasty. Twenty-one out of those 100 patients were treated with antibiotics, on a prophylactic basis, including the three patients that had infection. They were mild cases and easily treated. Yoder & Wiemert4 observed in a retrospective study postoperative infection in 0.48% of 1,040 patients, and none of them had been treated with antibiotics pre, intra or postoperatively.

Many otorhinolaryngologists also use topic formulations of antibiotics after nasal surgeries^4^^,^^10^. There is no evidence, however, that this practice reduces the incidence of postoperative infection^4^.

The indiscriminate use of antibiotics may also result in severe complications such as toxic reactions, reduction of the antibody formation stimuli, in addition to representing high costs and encouraging less strict compliance with good surgical practices^4^^,^^6^. The incidence of allergic reactions and antibiotics may vary from 0.7 to 10%, and fatal anaphylaxis occurs in 1 out of twenty-five thousand patients^7^.

## OBJECTIVES

The objective of the present study was to evaluate if it is necessary to use antibiotics in septoplasties, as well as major postoperative complications making a comparison between the use and no use of antimicrobial agents.

## MATERIAL AND METHOD

The study prospectively evaluated thirty-five patients that have undergone septoplasty associated or not with turbinectomy at Hospital das Clínicas, Medical School, University of Sao Paulo, from February to September 2004.

The protocol of the study was approved by the Ethics Committee for Research Project Analysis of the Clinical Board of Directors from Hospital das Clinicas, Medical School, University of Sao Paulo, under the research application # 133/04.

Selected patients were older than 15 years, and those patients with immunedeficiencies or any other sign of infection upon surgery were excluded, as well as those patients that had undergone previous nasal surgeries and presented nasal polyps or chronic sinusitis.

Patients were selected for surgeries based on clinical history, otorhinolaryngological exam and nasal endoscopy. Preoperative lab evaluation was performed in all patients using complete blood test, thrombin time, prothrombin time and thromboplastin time. Patients were evaluated by a hematologist before surgery if necessary.

All patients received general anesthesia and orotracheal intubation and signed the informed consent term related to the research protocol, surgical risks and likely complications resulting from the operation itself.

Patients were randomly divided into three groups according to the log number from the Hospital chart. Patients with chart number ending from 0 to 3 were included in group A, from 4 to 6 in group B, and from 7 to 9 in group C.

The first group (Group A) did not receive any type of antibiotics intra or postoperatively, the second group (Group B) received cephazolin 1.0 g IV only at anesthetic induction step, and the third group (Group C) received cephazolin 1.0 g IV at anesthetic induction step and cephalexin orally for 7 seven days postoperatively (500 mg every 6 hours).

Surgeries were performed by internists under guidance and supervision of assistant doctors of the Clinical Otorhinolaryngology Service, Clinical Hospital, Medical School – USP. The surgical technique used was endoscopy for turbinectomy and septoplasty using Cottle or Metzenbaun technique and photophore. Anterior nasal packing was applied in some cases.

An early postoperative protocol was applied, plus follow-up in the outpatient service after one week and after one month, investigating presence of bleeding events, fever (ancillary temperature above 380C), nausea, vomiting, pain, septal hematoma or abscess and purulent discharge in the nasal fossa.

Patients were medicated with symptomatic drugs (analgesic, fever and antihemetic drugs) if required, and graded pain from 0 (absence of pain) to 10 (maximum intensity) in order to objectively quantify it.

All patients underwent nasal endoscopy evaluation in the outpatient follow-up on the seventh and thirtieth day postoperatively. Endoscopic exam was always performed by the same professional that did not take part in the protocol and did not know if the patient was using antibiotics or not. This professional assigned a grade from 0 to 4 related to purulent discharge in the nasal fossa as follows: 0 (absence of secretion); 1 small amount, 2 moderate, 3 moderate to large amount, 4 massive.

Firstly, statistical analysis included the description of the sample patients investigated, using frequencies for category variables and mean and standard deviation for quantitative variables. The characteristics of the three groups of patients were compared to check if they were similar with use of Fisher Exact Test for category variables and variance analysis (ANOVA) for quantitative variables.

Secondly, complication incidence was compared among the three groups (category variables: Fisher Exact Test; quantitative variables: variance analysis with multiple comparisons, using Bonferroni's method). Statically analysis was carried out with STATA program version 8.0. Statistically significant values were considered as p < 0.05.

## RESULTS

The study included 35 patients as follows: sixteen patients did not take any antibiotics (Group A); eleven patients took it only upon anesthetic induction (Group B), and 8 at the induction step and for 7 days postoperatively (Group C).

In this group of 35 patients, 18 were female and 17 male patients and there was no gender-related difference between the groups (p = 0.56). The mean age of patients was 31.3 years (aged 15 to 58 years).

Mean age of group A was 30.75 years (standard deviation = 14.12), in group B it was 33.64 (SD = 13.90), and in group C it was 29.25 (SD = 15.27), therefore it was not statistically significant among groups (p = 0.79).

None of the patients developed hematoma or septal abscess postoperatively. Fever events also did not occur among patients.

Out of these 35 patients, there were 7 patients that left surgery room with anterior nasal packing (glove finger type), (Group A = 5 and Group B = 2) and there was not significant difference found between the two groups (p = 0.22). The other patients left surgery room without any type of packing.

In that group of patients without nasal packing, mild nose bleeding was reported postoperatively in 6 patients (17.14%), and in two of them anterior nasal packing was performed (5.7%, with glove finger in one and gel foam in the other patient) to control bleeding. The other four patients had easily controlled bleeding that improved by placing cold dressings over the face.

There was not any nausea and vomiting-related difference between the groups (Table 1) as well as postoperative pain (Table 2). None of the patients presented any complaints related to pain, nausea, vomiting in the follow up appointment on the 30th day postoperatively.

**Table 1:** Nausea & vomiting-related comparison between groups A, B, & C.

		1° PO				7° PO		
Symptom	Group A	Group B	Group C	p	Group A	Group B	Group C	p
	(N = 16)	(N = 11)	(N = 8)		(N = 16)	(N = 11)	(N = 8)	
Nausea	2 (12,5%)	0	1 (12,5%)	0,58	1 (6,25%)	0	0	1,0
Vomiting	2 (12,5%)	2 (18,2%)	0	0,66	1 (6,25%)	0	0	1,0

N = total number of cases. p = level of significance.

**Table 2:** Pain-related comparison between Groups A,B,C.

Pain (grade: 0 to 10*)		1° PO				7° PO		
	Group A	Group B	Group C	p	Group A	Group B	Group C	p
	(N = 16)	(N = 11)	(N = 8)		(N = 16)	(N = 11)	(N = 8)	
	Mean (SD)	Mean (SD)	Mean (SD)		Mean (SD)	Mean (SD)	Mean (SD)	
	2,31 (2,68)	0,91 (1,58)	0,75 (1,03)	0,13	0,31 (0,87)	0,73 (1,62)	0,37 (0,74)	0,63

N = total number of cases. p = level of significance, SD = Standard deviation

* subjective note assigned by the patient to pain with 0 = absence of pain and 10 = maximum intensity.

There were not any differences related to the amount of purulent discharge found in the nasal fossa through nasal endoscopy performed on the 7th and 30th day postoperatively (Table 3). None of the patients received grade 3 or 4 in nasal endoscopy on the 7th and 30th day postoperatively.

**Table 3:** Purulent discharge-related comparison in the nasal fossa among groups A, B and C.

		7° PO				30° PO		
Discharge purulent (grade*)	Group A	Group B	Group C	p	Group A	Group B	Group C	p
	(N = 16)	(N = 11)	(N = 8)		(N = 16)	(N = 11)	(N = 8)	
0	14 (87,5%)	8 (72,7%)	7 (87,5%)	0,44	14 (87,5%)	8 (72,7%)	7 (87,5%)	0,53
1	0	2 (18,2%)	0		2 (12,5%)	3 (27,3%)	1 (12,5%)	
2	2 (12,5%)	1 (9,1%)	1 (12,5%)		0	0	0	

N = Total number of cases. p = level of significance

* rating 0 (absence of discharge), 1 (small amount) and 2 (moderate).

## DISCUSSION

The first study about the prophylactic use of antibiotics in surgical procedures was conducted in 1938^6^, since then several management regimes have been proposed.

The most common reasons for prophylactic use according to a survey among the members of the US Society of Rhinology are “to prevent postoperative infection (60.4%), avoid toxic shock syndrome (TSS) (31.5%) and legal-medical aspects” (4.9%)^1^.

Currently, in turbinectomy, owing to the improvement of endonasal surgical techniques, postoperative nasal packing has become less used, reducing the infection risks. If septoplasty involves the use of nasal packing for 48 hours postoperatively, the risk of bacteremia is increased. Kaygusus^11^ et al. found bacteremia in 9 out of 53 patients (16.9%) after packing removal. Herzon12 observed bacteremia in 12% of 33 patients submitted to anteroposterior packing because of epistaxis.

The toxic shock syndrome is extremely rare, with estimated incidence of 0.0002% 1, and there is not any evidence that it could be prevented with prophylactic use of antibiotics.

Although surgical field (nasal fossa) is contaminated, bacteremia is not a relevant issue during nasal surgeries^5^^,^^7^^,^^12^, and if it occurs normally it is not likely to result in major clinical complications^11^. In one study carried out in 50 patients that have undergone septoplasty, although 46% of the patients had nasal mucosa colonized with staphylococcus aureus, none of the blood swabs collected during surgical procedures showed bacterial growth^5^.

Topic use of antibiotics also results in changes to the normal nasal flora, which is considered by many authors as a protective factor, favoring growth of gram-positive germs^12^.

Weimert et al.^6^ evaluated the postoperative interval of 174 patients that had undergone nasal surgeries, which were split into two groups. One treated with ampicillin 500mg 12 hours before surgery until 5 days after the procedures, and another group that did not take any antibiotics at all. Patients were evaluated through questionnaires and serial X-ray of paranasal sinuses and there were not any significant abnormalities between the groups concerning infection, scabs, bleeding, synechia, pain or ecchymoses. In this study, nasal fossa was not endoscopically evaluated.

The most common complication of nasal surgeries is hemorrhage, with incidence rate between 0.7 to 3.6 % of the cases 9. The second most common complication is infection. Most of infection-related complications occur in the surgical site, although they have been described as sinuses and intracranial complications, with thrombus of the cavernous sinus, meningitis, osteomyelitis and intracranial abscess. The absence of valves in facial, angular ethmoidal and ophthalmic vessels favors infection due to contiguity^9^. Local infections include cellulites, vestibulitis, septal abscesses and granulomas^9^.

In some special cases prophylactic use of antibiotics may be indicated, e.g. patients with cardiac valvulopathies or immunedepressed, in whom bacteremia may result in severe complications such as endocarditis, arthritis and osteomyelitis^7^^,^^11^.

There was no statistically significant difference among this group in this study concerning the amount of purulent discharge in the nasal fossa. None of the patients presented a large amount of purulent discharge (grade 3 or 4) in nasal endoscopy performed in the first week and in the first month postoperatively. Those patients with small amount (grade 1 or 2) were treated only with nasal lavage with abundant saline solution (0.9%), with good results and did not require use of antibiotics. The difference was not statistically significant among groups concerning nausea, vomiting, fever and pain postoperatively and after one week and one month follow-up.

## CONCLUSION

The incidence of nasal surgery complications is rare. Septoplasties are considered potentially contaminated surgeries, and do not require prophylactic use of antibiotics due to low risk of postoperative infection.
